# Couple’s concordance and discordance in household decision-making and married women’s use of modern contraceptives in Bangladesh

**DOI:** 10.1186/s12905-017-0462-3

**Published:** 2017-11-09

**Authors:** Jalal Uddin, Muhammad Zakir Hossin, Mohammad Habibullah Pulok

**Affiliations:** 1 Department of Sociology, University of Alabama at Birmingham, Birmingham, AL 35233 USA; 2 Department of Public Health Sciences, Karolinska Institute, Stockholm, Sweden; 3 Department of Economics, Southeast University, Dhaka, Bangladesh; 4 Center for Health Economics Research and Evaluation (CHERE), UTS Business School, University of Technology Sydney (UTS), Sydney, Australia; 5 CMCRC Health Market Quality Research Program, Sydney, Australia

**Keywords:** Couple, Modern contraceptives, Household decision-making, women’s power

## Abstract

**Background:**

Although a large body of studies documents that women’s autonomy in the household is associated with better reproductive health outcomes, these studies typically examined autonomy only from women’s point of view. The current study employs husband’s and wife’s perspectives together to examine the relationship between the decision-making arrangements in the household and the women’s use of modern contraceptives in Bangladesh.

**Methods:**

The study used the couple dataset of 2007 Bangladesh Demographic and Health Survey. The sample was comprised of 3336 married couples. Binary logistic regression models were used to examine the associations between the selected items on household decision-making and the use of modern contraceptives.

**Results:**

Our results indicate that the couples disagree considerably as to who in the household exercises the decision-making power. The pattern of decision-making regarding visiting family and relatives emerged as an important predictor of use of modern contraceptives in the multivariate regression analysis. The results suggest that compared to the couple’s concordant joint decision-making, the husband-only decision-making is associated with lower odds of contraceptives use (OR 0.49; 95% CI 0.28–0.85). Only a small part of this association is explained by spousal communication about family planning issues while the socio-demographic correlates hardly affected the association. On the contrary, the wife-only decision-making did not result in increased contraceptives use (OR 0.71; 95% CI 0.45–1.13).

**Conclusions:**

The study findings imply that women’s greater autonomy may not necessarily result in improved reproductive health behavior, and therefore, a balance of power in the spousal relationship is warranted.

## Background

Bangladesh has received considerable attention in the recent development literature as a surprising country for dramatic improvements in population, health and social development compared to countries with similar level of per capita income [1, 2]. Much of this attention is due to its rapid demographic transitions over the past four decades. Over the previous four decades, Bangladesh has experienced an eightfold increase in its contraceptive prevalence rate – from about 8% in 1975 to 62% in 2014, and rapid decline in total fertility rates - from about 7 births per woman in the 1970s to 2.3 births per woman in 2014 [3]. These achievements are largely attributed to a high contraceptive acceptance rate in general and especially women’s use of modern contraceptives.

Despite being a low-income and Muslim-majority country with traditional gender system, women’s social status and access to economic spheres have considerably increased in the recent decades in Bangladesh. One of the most visible changes in women’s lives has been the increased employment, particularly in the export-oriented garment sector. This has also happened in private and public enterprises. A recent study demonstrates that young girls exposing to ready-made garment sector can substantially delay marriage and childbirth [4]. In addition, donor assisted development programs such as the widespread use of microfinance programs for poor women, provision of stipend for female education, women’s easy access to community-based maternal and child health services have significantly contributed to alter the prevailing gender roles and break the silence of womenfolk. All of these factors inherently allow women greater economic power and autonomy in the family [5, 6]. A substantial body of existing literature shows that women’s power and autonomy is favorably related to better maternal and reproductive health and family planning outcomes [7–13]. These studies commonly provide evidence for one compelling proposition that women who exhibit substantial autonomy in the household have greater ability to control their body and achieve desired fertility and reproductive goals.

Most of the previous studies have primarily measured women’s decision-making power using only women’s reports [14]. However, decisions about adopting family planning such as using contraceptives for either spacing or liming childbirth are often strongly shaped by spousal relationships [15]. As a result, a growing number of studies attempt to employ a couple approach which examines responses from both wives and husbands. One advantage of using a couple approach is that it can explore the couple level discrepancies between men’s and women’s reports of knowledge, attitudes, and behaviors related to fertility, family planning, and reproductive healthcare [16]. Lately, ‘couples studies’ have examined the association between various health behavior outcomes and couples’ responses of their household decision-making [15–18]. These studies tend to use women’s participation in household decision-making as a proxy measure of their relative power and autonomy in the family. These studies measured decision-making using varying reports obtained from different groups of respondents (i.e., wife, husband, and couple).

Existing literature on determinants of family planning adoption extensively focused on socioeconomic, demographic, spousal communication about family planning, and family planning program access factors [19–26]. Some studies looked at the effects of women’s autonomy measures, such as household decision-making measured through the use of only women’s reports of household decision-makings [27–31]. These studies largely overlooked the concordance or discrepancy between husbands’ and wives’ reports about women’s autonomy and the extent to which such concordance or discrepancy may account for differences in women’s use of contraceptives. Using the Bangladesh Demographic and Health Survey’s couple sample, our study examines the concordance and discordance in married couple’s decision-makings by matching husbands’ and wives’ responses to a set of household decision-making questions. Furthermore, controlling for previously reported socio-economic and family planning program access factors related to contraceptive use, this study explores the association between different decision-making arrangements as reported by both partners and women’s current use of modern contraceptive methods at multivariate level.

## Methods

### Data and sample

The study used secondary data from the 2007 Bangladesh Demographic and Health Survey (BDHS). This is a nationally representative household survey in Bangladesh conducted in every 3 years. Based on a multi-stage stratified random sampling technique, the 2007 BDHS conducted face-to-face interviews among the ever-married women and men in the 10,819 selected households. A total of 10,996 women of reproductive age (15–49) participated in the survey, yielding a response rate of 98.4%. On the other hand, the male participants comprised a subsample of the sampled households, with 3771 men aged 15–54 years interviewed from every second household with a response rate of 92.6%. The data collection and sampling procedure have been described in details elsewhere [32]. The analytic sample in the present study, however, is restricted to 3336 currently married couples where complete information on all variables of interest from both partners was available.

### Outcome variable

The outcome of interest is a binary variable of whether women were currently using any modern contraceptive method at the time of the survey. Women were categorized as users of any modern method if they were using any of the following methods: oral pills, intrauterine devices (IUDs), injections, condoms, sterilization, and implants (norplant). Women not using any of the above contraceptive methods or using periodic abstinence, withdrawal, or other traditional methods were categorized as not using any modern method.

### Main predictors

The main predictors of the outcome variable are the measures of concordance and discordance between the husband and wife about household decision-making. In the 2007 BDHS, both the husband and the wife were asked who in the household makes decisions regarding the following: i) major household purchases, ii) purchases of daily household needs, and iii) visits to their families or relatives. Unlike most other prior studies on couple’s decision-making that constructed a summary index [18, 33], our study drew on the work of Story and Burgard [16] and separately used the individual decision-making items to measure the level of concordance and discordance in the responses of the husband and the wife. There were five response alternatives for each of the decision-making questions: 1) wife only, 2) husband only, 3) wife and husband jointly, 4) someone else, and 5) respondent and someone else. The last two categories were collapsed into the ‘other’ category in our analyses. We then cross-tabulated the responses of the husband and the wife to assess the level of spousal concordance and discordance and introduced the following five categories into the multivariate regression analyses: 1) agree - wife only, 2) agree - husband only, 3) agree - jointly, 4) agree - other, and 5) disagree. Thus, the first four categories reflect spousal concordance about who decides in the household while the fifth category - disagree - was introduced to capture the whole amount of discordance across all response categories. ‘Agree - jointly’ is used as the reference category in the regression models.

### Other covariates

In addition to the three decision-making items, a number of explanatory variables have also been chosen on a priori knowledge [7, 10, 11, 16–18, 34]. These include the socio-demographic characteristics of the study subjects, particularly of the women; husband-wife communication about family planning issues; and exposure to family planning information. Communication about family planning was measured by asking the respondents about how often they talked about it with their spouse within 3 months preceding the survey and the response alternatives were: never, once or twice, and more often. Exposure to family planning was assessed by asking if the respondents had ever heard about it on television or radio and measured with binary options (no/yes). Among the socioeconomic variables, household wealth status was assessed by creating a wealth index. Based on the selected assets owned by a household and the dwelling characteristics, each household was given a weight generated through principal component analysis. The summed scores for each household were then used to rank the individuals by dividing the sample into five quintiles representing five distinct hierarchical groups: poorest, poorer, middle, richer, and richest [35, 36]. Other socio-demographic factors included in the analysis were the wife’s age at survey (used as a continuous variable), parity or the number of living children (grouped as none, 1–2, ≥3), place of residence (rural, urban), religion (Muslim, other), wife’s education (no education, primary, secondary, or above), and wife’s employment status (no, yes).

### Statistical analyses

The associations between household decision-making arrangements and use of contraceptives were first examined at the bivariate level to see whether the observed associations vary depending on the responses given by the wife or the husband or the paired responses from both partners. Multivariate logistic regression was used to estimate the adjusted odds ratios (ORs) for the underlying associations between the concordant and discordant responses of the couples and contraceptive use, controlling for other explanatory variables. Four models were estimated: the first model included couple’s concordant and discordant decision-making responses, the second model included husband-wife communication about family planning, and the third model further adjusted for exposure to family planning. In the final model, adjustment was made for selected socio-demographic correlates.

## Results

Table 1 presents the socio-demographic profile of the study participants. The average age of the women in this sample was approximately 30 years with a standard deviation of 8.5. Most of the couples had children with 48% having three or more living children while 9% were childless. Majority of the couple lived in rural areas (62.3%) and more than two thirds (70%) were unemployed. Around 29% of the women had no schooling at all and almost 40% had attained secondary or higher level of education. The highest proportion of couples belonged to the richest wealth quintile (26%) while 16% were in the poorest quintile. The proportion of couples belonging to the poorer, middle, and richer quintile was roughly similar (around 19%). As regards the exposure to family planning information, 33% heard about it on television and 12% on radio. Besides, almost half of the participants reported that there was no communication with their husbands about family planning within 3 months prior to the survey.

**Table 1:** Socio-demographic characteristics of the sample

	N	Percent
Wife’s age	3336	Mean = 29.56 (SD = 8.55)
Parity		
0	310	9.29
1–2	1436	43.05
≥ 3	1590	47.66
Residence		
Rural	2077	62.26
Urban	1259	37.74
Wife’s religion		
Islam	2968	88.97
Other	368	11.03
Wife’s education		
No education	958	28.72
Primary	1049	31.44
Secondary or above	1329	39.84
Wife’s employment		
No	2338	70.1
Yes	997	29.9
Household economic status		
Poorest	546	16.37
Poorer	643	19.27
Middle	657	19.69
Richer	638	19.12
Richest	852	25.54
Exposure to FP information		
No exposure	1830	54.16
Heard FP on radio	408	12.23
Heard FP on television	1098	32.91
FP communication in last 3 months		
Never	1631	48.92
Once or twice	1335	40.04
More often	368	11.04

Table 2 demonstrates the couple’s matched responses with regard to their mode of participation in three major household decisions and their concordance and discordance in this respect. The concordant reports, as shown in the table, reveals that the joint involvement of both husband and wife is the predominant decision-making arrangement across all three questions (ranging from 17% to 35%) whereas the woman’s single-handed decision over the concerned household affairs was the least common practice. As regards the one-sided decision-making arrangement where either the wife or the husband alone makes the decisions, a gender difference seems to exist in proportion to participation in all three decision-making items measured in this study. Whereas the average number of wives who decide alone on the household affairs ranges from 0.3% to a maximum of 4% depending on the type of household decision, the corresponding representation of the husbands deciding alone varies between 8% and 9%. Overall, the greatest magnitude of spousal concordance is observed with regard to major household purchases (51%) whereas the decision to purchase daily household necessities yielded the least spousal agreement (37%).

**Table 2:** Cross-tabulation of couples’ responses to all three decision-making items

Wife’s response	Husband’s response				Total
	Wife	Husband	Jointly	Other	
Who usually makes decisions about making major household purchases?					
Wife	**0.34**	1.49	3.26	0.28	5.37
Husband	0.49	**8.53**	15.69	3.35	28.07
Both Jointly	1.25	12.36	**34.18**	4.73	52.52
Other	0.09	2.29	3.23	**8.42**	14.04
Total	2.18	24.68	56.37	16.78	100
Spousal agreement = 51.47%					
Who usually makes decisions about making purchases for daily household needs?					
Wife	**4.08**	10.22	14.37	1.79	30.46
Husband	1.94	**8.92**	8.98	1.35	21.18
Both Jointly	4.34	12.06	**17.25**	1.63	35.28
Other	0.55	3.29	2.33	**6.91**	13.08
Total	10.91	34.5	42.92	11.68	100
Spousal agreement = 37.16%					
Who usually makes decisions about visits to your family or relatives?					
Wife	**0.52**	1.5	5.45	0.35	7.82
Husband	0.99	**7.87**	17.14	3.08	29.08
Both Jointly	1.44	11.88	**35.08**	3.19	51.59
Other	0.09	2.2	3.72	**5.48**	11.5
Total	3.04	23.46	61.4	12.11	100
Spousal agreement = 48.95%					

The figures in boldface show the percentage points of concordance in each categories

A substantial amount of spousal discordance is also found in the couple’s reports on who decides what in the family. In the wife-arranged type of decision-making, wives are more likely to refer to themselves as the sole decision makers than what the husbands report about their wives’ sole involvement in the decision-making process. For instance, over 30% of the women reported that they decide about purchasing the daily household necessities in contrast to the husbands’ report of 11%. On the other hand, the women consistently tended to underestimate the joint decision-making arrangement when compared to men. For example, while 61% of men reported that they jointly decide when visiting the wife’s family or relatives, the corresponding figure for women amounts to 52%. A mixed pattern of discordance is, however, found in the husband’s reports about decision-makings.

Table 3 shows the associations between the types of decision-making within the household and the current use of modern contraceptives at the bivariate level. The analyses found that the husbands’ one-sided decisions in all three decision-making items are associated with lower odds of use of modern contraceptives, although two out of three decision-making items show non-significance when only husbands’ reports are considered. A strong negative association, in general, is found between the ‘other’ type of decision-making category and the use of contraceptives. For example, according to the couple’s report, compared to a spouse who jointly makes a decision about purchasing the daily household necessities, the odds of using contraceptives is 41% lower (OR 0.59; 95% CI 0.43–0.81) among the spouses who have little or no say in the decision. On the other hand, in none of the decision-making items did the wife-only decision-making show any statistically significant relationship with contraceptive use. The bivariate analyses further reveal that spousal disagreement has a strong negative association with use of contraceptives in decision-making item concerning the major household purchases (OR 0.79; 95% CI 0.65–0.96).

**Table 3:** Bivariate logistic regression analysis of the association between couple’s decision-making arrangement and wife’s current use of modern contraceptive

Decision-making items	Wives		Husbands		Couples	
	OR	95% CI	OR	95% CI	OR	95% CI
Who decides about making major household purchases?						
Jointly (Reference)						
Wife Only	1.06	[0.74–1.53]	0.71	[0.46–1.10]	1.03	[0.30–3.58]
Husband Only	0.75***	[0.61–0.91]	0.87	[0.71–1.06]	0.76*	[0.55–1.05]
Others	0.62***	[0.49–0.78]	0.74***	[0.60–0.92]	0.62***	[0.47–0.84]
Disagree					0.79**	[0.65–0.96]
Who decides about making daily household purchases?						
Jointly (Reference)						
Wife Only	1.03	[0.86–1.23]	0.92	[0.70–1.21]	0.96	[0.62–1.48]
Husband Only	0.80**	[0.65–0.99]	0.83**	[0.69–0.99]	0.73*	[0.52–1.01]
Others	0.62***	[0.49–0.78]	0.64***	[0.50–0.82]	0.59***	[0.43–0.81]
Disagree					0.97	[0.77–1.22]
Who decides about visits to your family or relatives?						
Jointly (Reference)						
Wife Only	1.09	[0.83–1.44]	0.92	[0.63–1.35]	1.80	[0.62–5.25]
Husband Only	0.77***	[0.63–0.93]	0.87	[0.71–1.07]	0.73**	[0.53–1.00]
Others	0.59***	[0.46–0.76]	0.72***	[0.57–0.92]	0.56***	[0.41–0.77]
Disagree					0.93	[0.77–1.12]

Significance level ***p < 0.01, **p < 0.05, *p < 0.10

The multiple regression analyses presented in Table 4 demonstrate the strengths of associations between decision-making arrangements and the use of contraceptives adjusting for a wide array of covariates. In model one, the husband’s autonomy and spousal disagreement are associated with lower prevalence of contraceptive use as far as the decision to visit wife’s home or relatives is concerned. Thus, compared to concordant joint decision-making, the estimated odds of using contraceptives is 51% lower (OR 0.49; 95% CI 0.28–0.85) when the husband alone makes the decision. A statistical adjustment of husband-wife communication about family planning issues, however, attenuated the original associations to some extents. For example, 23.5% [(0.61–0.49)/(1–0.49) *100] of the main association between ‘husband only’ decision-making and contraceptive use is explained by spousal communication about family planning affairs indicating that the latter is also an important predictor of the use of contraceptives. When exposure to family planning information is adjusted for, the effect sizes remained just as large. Controlling for socio-demographic factors hardly changed the association between husband only decision-making and contraceptive use, but the association with spousal disagreement weakened considerably and turned out to be marginally significant. Thus, when all other factors are held constant, the husband’s unilateral decision to visit the wife’s family or relatives lowers the chance of using modern contraceptives by 38% (OR 0.62; 95% CI 0.36–1.07) compared to a household setting where both husband and wife jointly make the decision. However, contrary to the widely held belief about the relationship between women’s autonomy and increased use of contraceptives, women’s autonomy in the present study showed no significant association at all.

**Table 4:** Multivariate logistic regression on the association between couple level decision-making arrangement and wife’s current use of modern contraceptive controlling for FP exposure and socio-demographic factors

Variables	Model 1		Model 2		Model 3		Model 4	
	OR	[95% CI]	OR	[95% CI]	OR	[95% CI]	OR	[95% CI]
Who decides about making major household purchases? Agree (Jointly = ref)								
Agree - Wife only	1.09	[0.69–1.73]	1.08	[0.68–1.70]	1.05	[0.67–1.66]	0.96	[0.60–1.54]
Agree - Husband only	0.96	[0.67–1.39]	1.10	[0.74–1.63]	1.11	[0.75–1.64]	1.09	[0.73–1.64]
Agree – Other	0.87	[0.57–1.32]	0.91	[0.60–1.38]	0.88	[0.58–1.33]	1.07	[0.67–1.72]
Disagree	1.13	[0.87–1.46]	1.17	[0.87–1.56]	1.17	[0.88–1.56]	1.15	[0.85–1.56]
Who decides about making daily household purchases? Agree (Jointly = ref)								
Agree - Wife only	2.16	[0.74–6.31]	2.47	[0.64–9.54]	2.62	[0.67–10.26]	2.53	[0.67–9.49]
Agree - Husband only	1.01	[0.71–1.43]	1.08	[0.75–1.56]	1.10	[0.76–1.59]	1.12	[0.76–1.65]
Agree – Other	0.86	[0.53–1.39]	0.68	[0.42–1.08]	0.70	[0.44–1.11]	0.88	[0.51–1.52]
Disagree	1.13	[0.91–1.39]	1.16	[0.91–1.47]	1.18	[0.92–1.50]	1.20	[0.94–1.55]
Who decides about visits to your family or relatives? Agree (Jointly = ref)								
Agree - Wife only	0.71	[0.45–1.13]	0.82	[0.52–1.28]	0.82	[0.52–1.29]	0.81	[0.52–1.26]
Agree - Husband only	0.49**	[0.28–0.85]	0.61*	[0.35–1.05]	0.61*	[0.35–1.05]	0.62*	[0.36–1.07]
Agree – Other	0.56	[0.27–1.14]	0.76	[0.37–1.57]	0.75	[0.36–1.58]	0.62	[0.28–1.37]
Disagree	0.61***	[0.51–0.74]	0.70***	[0.58–0.86]	0.70***	[0.57–0.86]	0.84	[0.68–1.04]
Husband-wife communication about FP in last 3 months (Never = ref)								
Once or twice			4.66***	[3.84–5.66]	4.62***	[3.81–5.60]	4.38***	[3.57–5.38]
More often			4.22***	[3.05–5.83]	4.17***	[3.02–5.77]	4.13***	[2.90–5.87]
Exposure to FP information(No = ref)								
Heard FP on radio					0.77*	[0.59–1.01]	0.86	[0.64–1.15]
Heard FP on TV					1.24**	[1.00–1.53]	1.18	[0.94–1.48]
Wife’s age							0.98***	[0.96–0.99]
Parity (0 = ref)								
1–2							6.56***	[4.64–9.26]
≥ 3							9.05***	[6.06–13.50]
Urban residence (Rural = ref)							1.11	[0.88–1.41]
Other religion (Muslim = ref)							1.03	[0.66–1.60]
Wife’s education (No education = ref)								
Primary							1.06	[0.83–1.35]
Secondary or above							1.05	[0.81–1.37]
Wife’s employment (No = ref)							1.31**	[1.06–1.61]
Household economic status (Poorest = ref)								
Poorer							1.04	[0.75–1.44]
Middle							0.99	[0.74–1.33]
Richer							1.20	[0.84–1.73]
Richest							1.36	[0.93–1.99]

Significance level: ***p < 0.01, **p < 0.05, *p < 0.10

The multiple logistic regression further indicates that, of all the variables included in the fully adjusted model, parity and spousal communication emerged as the strongest predictors of contraceptive use among the study subjects. Thus, compared to the women with no children, women having three or more children have a significantly much higher likelihood of using contraceptives (OR 9.05; 95% CI 6.06–13.5). Similarly, the regular spousal communications about family planning issues increase the odds of contraceptive use by a factor of 4.13 (95% CI 2.90–5.87). Moreover, older women are less likely to use contraceptives than younger women, with every one-year increase in age being associated with a 2% significant decline in the odds of contraceptives use (OR 0.98; CI 0.96–0.99). Among the employed women, the odds of contraceptives use are 1.31 times higher compared to those who are unemployed (95% CI 1.06–1.61).

## Discussion

This study contributed to the growing body of couple studies at least in two ways. First, unlike existing studies that predominantly use women’s reports in measuring decision-making power, we used a couples’ perspective to measure how couples view their participation in the household decision-making process. Second, by using measures of couple’s concordance and discordance in household decision-making, the study provided empirical evidence that there are gender-based differences in spouses’ reports of their participation in household decision-making. We also found that couples significantly vary in reporting how decisions are made in the family (e.g. husband only, wife only, jointly, etc.), and individual respondent’s (e.g. husband, wife) and paired couple’s reports have different influences on women’s use of modern contraceptives.

The analysis revealed several methodologically critical and policy-relevant findings. First, though spousal agreement dominates across all decision-making items, there are also considerable amounts of discrepancies between husbands’ and wives’ reports about who usually makes household decisions. Second, decisions made by the husband alone, or involvement of other family members in the decision-making process tend to be associated with lower likelihood of use of any modern contraceptives. Third, women’s unilateral decision making is unlikely to have a positive influence on contraceptive use. Finally, unlike previous studies [29–31] that highlighted the salient role of women’s sole autonomy in fertility and reproductive health outcomes, our analyses point to the idea that couples who upheld the shared decision-making ideals and demonstrate egalitarian gender relations seemed to be more likely to use contraceptives.

The multivariate analysis finds that compared to concordant joint decision-making category (e.g. reference category in the regression models), statistically significant coefficients in other categories were consistently lower. The study findings, moreover, suggest that negotiation and discussion about household affairs between husbands and wives is instrumental in pursuing common interests such as family planning. Such findings support the argument that increased spousal consultation and negotiation enables couples in overcoming conflicting preferences and goals about family issues including fertility regulation [37]. Historically, within the domain of intra-household gender relationship, women’s identity and status are structurally tied to men in systems of kinship and marriage in the South Asian context, where ideals of togetherness and co-operated interdependence are highly emphasized [38, 39].

Broadly, our findings call into question the individualistic autonomy paradigm that highlights women’s independent and autonomous position in the family while putting the entire burden of responsibility on them to pursue actions for private concerns. However, researchers often suggest that autonomy framework is inappropriate in the context where women’s status is structurally tied to men. Studies that examined the gender-based power relations in South Asian context show that spouses in the household are often tied together by strong emotional and structural bonds and married women’s status is embedded in social relationship and kinship ties [38–40]. These studies highlight that autonomy framework undermines the view that support from significant others may, in fact, benefit women’s use of shared resources and uptake of needed health services. A growing body of research suggests that men’s involvement in the decision-making process and antenatal health education programs promote better partnership between spouses in the household and women’s maternal healthcare-seeking behavior in poor settings such as rural Guatemala and Nepal [41, 42].

The study results show that involvement of husband alone or others in decisions particularly concerning the visit to family or relatives is associated with lower contraceptive use compared to concordant joint decision-making. Such findings may suggest that when husbands or other family members solely control women’s freedom of mobility, it adversely affects women’s use of contraceptive. It is partly because women’s freedom to move, when controlled by husband or other family members, may restrict their access to reproductive health resources and information. In this regard, it is worth mentioning that husbands’ and other family members’ authority to control women’s physical mobility may result from the Muslim institution of *purdah* in Bangladesh*.* The usual practice of *purdah* includes restrictions on women’s physical movement outside the home, and enforcement of covering themselves when stepping into public spheres, with the purpose to hide them, especially from non-kin members. Evidence suggests that such secluding practices often limit women’s opportunities to participate in income-generating activities and access to reproductive healthcare services [43–45].

It is interesting to notice that, of the three decision-making items, only one item that relates to women’s freedom of mobility was significantly associated with the outcome variable in the multivariate analysis. Such findings warrant some discussion regarding the relative importance of different dimensions of women’s autonomy and the context in which these dimensions are applied. Women’s autonomy is a multi-dimensional concept, which captures multi-faceted ability to have control over one’s strategic life choices [46]. The concept of autonomy is typically measured by using items representing several dimensions of autonomy such as control over material/financial resources, household decision-making, freedom of movement, and freedom from violence. The decision-making item that relates to women’s freedom of mobility is particularly interesting in the context of Bangladesh, where patriarchal cultural practice such as *purdah*, although gradually declining, is still socially valued, especially in the rural settings. The non-significance of other decision-making items such as decisions about daily household purchases can plausibly be explained by the argument that not all decision-making items are strategically important for women’s autonomy and power. In many settings, decisions such as what to cook, small household purchases, and family food budget, etc. are traditionally made by women. Likewise, feminist scholars often suggest that selection of indicators of autonomy should either be context specific or theoretically guided [47]. Therefore, an indicator of freedom of mobility may be most sensitive and relevant in a patriarchal context such as Bangladesh, where female seclusion is still socially valued in stark contrast to the western context [48].

Apart from the decision-making measures, spousal communication about family planning emerged as a strong predictor of contraceptive use, supporting previous literature that shows that [19, 20, 24, 41] communication between husband and wife about family planning is significantly associated with increased use of contraceptives. The large size of the odds ratios related to this variable and the attenuation of the main association after adjusting for it indicate that husband-wife communication about family planning is one of the most direct and cardinal predictors of contraceptive use. Evidence suggests that frequent couple’s discussion and approval of family size have a significant independent effect on contraceptive use [22, 41]. On the other hand, lack of spousal communication about family planning and limited negotiation for reproductive preferences negatively affects the consistent use of contraceptive. Further, consistent with the previous studies [20, 21, 27], our analysis finds that parity is positively related to contraceptive use. This simply implies that as high parity women tend to achieve their fertility goals, they are more likely to limit childbirth and use contraceptives.

However, the findings should be interpreted bearing several limitations in mind. One major limitation is that we cannot infer any causal relationship between decision-making items and contraceptive use because of the cross-sectional nature of the data. Decision-making processes are complex and dynamic and previous experiences over time may shape couple’s current patterns of decision-making. Further, the use of a limited number of decision-making questions may seem to be an issue of content validity. Although BDHS collects information on a broad range of questions on household decision-making from the women sample, the couple datasets available contain a limited number of questions comparable between spouses. It is likely that if we could use some other decision-making items (e.g. who decides about healthcare, who controls the money women/men earn, who decides the number of children to have, and who decides the arrangement of children’s marriages), we could have found a clearer picture of the association under investigation. Studies using a large number of such decision-making items found that a broader range of decision-making items strongly predict adult health and antenatal care utilization behavior [40, 49]. Therefore, future couple studies may develop and use a broader array of decision-making questions capturing different dimensions of autonomy and examine their association with family planning outcomes.

Additionally, more work is needed to examine the associations in relation to the use of contraceptive by both husbands and wives, not just wives’ use of contraceptive and in relation to various types of contraceptives. In this regard, it is worth mentioning that women’s decision-making power might have different implications for the use of different types of contraceptives, especially the use of contraceptives by men. In Bangladesh, the use of male condom accounts for only 11% of all modern methods. On the other hand, women predominantly use the short-term methods such as pills and injectables, which account for about 75% of all modern methods [3]. The current family planning policies in Bangladesh highlight the need to promote the use of long-acting methods such as intrauterine devices or sterilization, although there has not been great demand for such methods [3]. Future studies can examine whether couple’s decision-making power dynamics may predict the use of more permanent and long-acting reversible contraceptives and perhaps gender differences in the use of long-acting methods. Despite these limitations, the key strength of this study lies in the fact that couple’s matched reports give us perhaps the most reliable estimates of what is happening in the intra-household power practices in Bangladesh.

## Conclusions

Using responses of married couples, this study demonstrated that couple’s concordance or discordance in household decision-making has a considerable bearing on predicting women’s use of contraceptives in Bangladesh. Therefore, conclusions should be drawn based on both husband’s and wife’s reports since the data based on wife’s /husband’s reports alone may not accurately capture the true phenomenon, potentially leading to biased estimates. From a policy perspective, the study findings suggest that promoting a balance of power between the husband and the wife is possibly more effective than promoting women’s autonomy when it comes to increasing the prevalence of contraceptives use. Policy makers in family planning and reproductive healthcare should focus on interventions and program efforts targeting the imbalanced power practices in the household.
